# Efficacy of unregulated minimum risk tick repellent products evaluated with *Ixodes scapularis* nymphs in a human skin bioassay

**DOI:** 10.1186/s13071-024-06146-3

**Published:** 2024-02-01

**Authors:** James C. Burtis, Shelby L. Ford, Christina M. Parise, Rebecca J. Eisen, Lars Eisen

**Affiliations:** Division of Vector-Borne Diseases, Centers for Disease Control and Prevention, Fort Collins, CO USA

**Keywords:** *Ixodes scapularis*, Repellents, Natural repellents, Essential oils, Duration of repellency

## Abstract

**Background:**

The majority of vector-borne disease cases in the USA are caused by pathogens spread by ticks, most commonly the blacklegged tick, *Ixodes scapularis*. Personal protection against tick bites, including use of repellents, is the primary defense against tick-borne diseases. Tick repellents registered by the Environmental Protection Agency (EPA) are well documented to be safe as well as effective against ticks. Another group of tick repellent products, 25(b) exempt or minimum risk products, use alternative, mostly botanically derived, active ingredients. These are considered to pose minimal risk to human health and therefore are exempt from EPA registration; efficacy testing is not mandated for these products.

**Methods:**

We used a finger bioassay to evaluate the repellency against *I. scapularis* nymphs for 11 formulated 25(b) exempt products together with two positive control DEET-based EPA registered products. Repellency was assessed hourly from 0.5 to 6.5 h after product application.

**Results:**

The DEET-based products showed ≥ 97% repellency for all examined timepoints. By contrast, an average of 63% of ticks were repelled in the first 1.5 h after application across the 11 25(b) exempt products, and the average fell to 3% repelled between 2.5 and 6.5 h. Ten of the 11 25(b) exempt products showed statistically similar efficacy to DEET-based products at 30 min after application (repellency of 79–97%). However, only four 25(b) exempt products maintained a level of repellency similar to DEET-based products (> 72%) at the 1.5-h mark, and none of these products were effective in repelling ticks at the timepoints from 2.5 to 6.5 h after application.

**Conclusions:**

Neither the claims on the labels nor specific active ingredients and their concentrations appeared to predict the duration of efficacy we observed for the 25(b) exempt products. These products are not registered with the EPA, so the methods used to determine the application guidelines on their labels are unclear. Consumers should be aware that both the level of efficacy and the duration of repellency may differ among unregulated 25(b) exempt repellent products labeled for use against ticks. We encourage more research on these products and the 25(b) exempt active ingredients they contain to help determine and improve their efficacy as repellents under different conditions.

**Graphical Abstract:**

**Figure:**
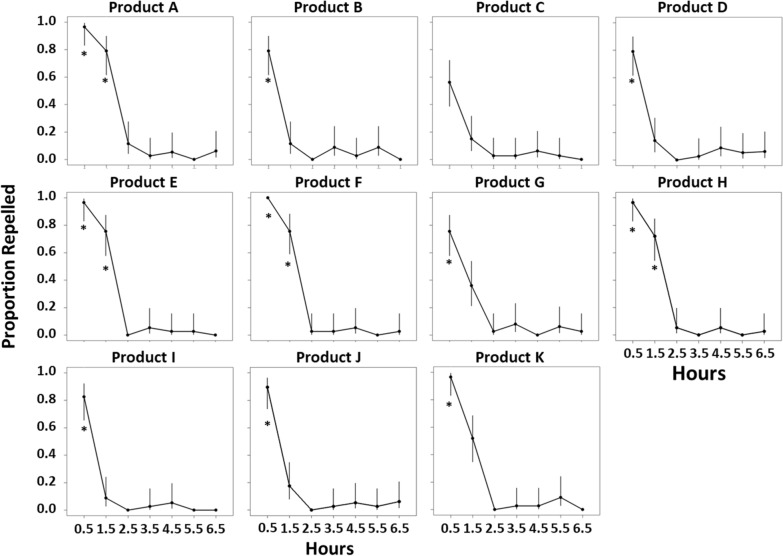

## Background

Geographic range expansion has been documented over the last 2 decades for multiple tick species of medical concern in the USA, including *Ixodes scapularis* (blacklegged tick), *Amblyomma americanum* (lone star tick), and *A. maculatum* (Gulf Coast tick) [1]. This has placed an increasing number of people at risk for the disease agents and medical conditions associated with bites by these tick species [2]. There are currently no vaccines for humans against tick-borne pathogens in the US, and control of ticks in the environment has proven to be very challenging [3]. Therefore, use of personal protection measures, including repellents and permethrin-treated clothing, remains the first line of defense against tick bites and tick-borne disease.

The use of repellents to prevent tick bites is recommended by the Centers for Disease Control and Prevention (CDC) as well as state health agencies in the US. However, repellent use is limited even in the parts of the US with the highest incidence of tick-borne disease. For example, a recent survey showed that only 25% of respondents in states with high Lyme disease incidence used tick repellents routinely [4]. Commercially available tick repellents include products registered by the Environmental Protection Agency (EPA) as well as unregulated minimum risk or 25(b) exempt products. EPA product registration requires demonstration of the product’s safety and effectiveness. By contrast, minimum risk products are exempt from EPA registration because the ingredients they contain are deemed safe for humans and the environment [5]; demonstration of effectiveness is not mandated. Due to their well-documented safety and effectiveness, CDC recommends using EPA-registered tick repellent products that contain at least one of the following six active ingredients: DEET, picaridin, IR3535, oil of lemon eucalyptus, para-menthane-diol, and 2-undecanone [6]. The EPA provides a searchable directory of these products, including information for the number of hours they are expected to effectively repel ticks after application [7].

Unregulated 25(b) exempt minimum risk repellent products are most commonly based on essential oils, and marketed as natural products, which may be appealing to consumers. However, there are concerns about the efficacy of these formulated products to protect against tick bites as published data from laboratory studies using human skin assays to evaluate their repellency are lacking [8]. Moreover, application guidelines on the labels of these products vary broadly, which can make it difficult to determine how they should be used. Although published studies on formulated products are lacking, a recent study tested the efficacy of a wide variety of unformulated 25(b) exempt active ingredients in the form of essential oils (10%) as repellents for *I. scapularis* adults in a human skin bioassay [9]. Many of these essential oils effectively repelled ticks for 60 min, with some providing protection up to 112 min, whereas a positive DEET control repelled ticks for the full 6-h evaluation period. Another recent study investigating the repellency of two unformulated active ingredients (20% peppermint and rosemary oils in ethanol) and a positive control (DEET) yielded similar results in a human skin bioassay with *I. scapularis* nymphs; repellency of the essential oils was absent (rosemary oil) or short-lived (< 120 min for peppermint oil), whereas DEET repelled ticks for 6 h [10].

The limited duration of efficacy shown when 25(b) exempt active ingredients are tested individually may not reflect the efficacy of the formulated repellent products on the market. Most commercial 25(b) exempt repellent products utilize more than one active ingredient, which may synergize to enhance efficacy. These products also contain inert chemical compounds that might act as stabilizers or synergists to further enhance the repellent activity. To start addressing the lack of published data for 25(b) exempt minimum risk tick repellent products, we evaluated the repellency of selected products in a human skin bioassay against *I. scapularis* nymphs hourly from 0.5 to 6.5 h after application, with DEET-based EPA-registered products as positive controls. *Ixodes scapularis* nymphs were used because this species commonly bites humans [11] and the nymphs are considered the primary vectors to humans of multiple pathogens, including bacterial agents causing Lyme disease and anaplasmosis, a parasite causing babesiosis, and the virus causing Powassan encephalitis [12].

## Methods

### Tick rearing

The *I. scapularis* nymphs used in our bioassay were derived from a colony maintained at the CDC, Division of Vector-Borne Diseases (DVBD) in Fort Collins, CO, USA. This *I. scapularis* colony is occasionally refreshed with field-collected adults from the northeastern US, most recently in the spring of 2023 with males from Rhode Island. Larvae and nymphs are fed on CD1 mice (Charles River Laboratories, Wilmington, MA, USA), while adults are fed on New Zealand white rabbits (Charles River Laboratories). Animals were used for this purpose under approved protocols on file with the DVBD Institutional Animal Care and Use Committee. All tick life stages are maintained in incubators maintaining a 16:8 h light:dark cycle at 24 °C in desiccators containing a potassium sulfate solution (120 g/l) to maintain 90–95% humidity. Nymphs were used in the bioassays 2–5 weeks after molting from larvae.

### Repellent product selection

The 25(b) exempt formulated repellent products tested were selected by reviewing three popular commercial websites (Amazon, Target and Walmart). A search for ‘natural tick repellent’ was conducted across all three websites. We also selected products by searching specific categories, which differed on each website. On Amazon, products listed in the ‘tick repellent’ and ‘insect & pest repellent body sprays’ sections were included in the search. For Target, we searched the ‘insect and pest control’ section. For Walmart, we searched the ‘insect and pest repellents’ section. For a repellent product to be included, it needed to specifically mention repellency against ticks on the label, be targeted for use on human skin, and contain at least one 25(b) exempt active ingredient. EPA-registered products containing active ingredients DEET, picaridin, IR3535, oil of lemon eucalyptus, para-menthane-diol, or 2-undecanone were not included as natural products. Reviews for each product were also examined. Only those with at least four out of five stars based on at least 100 reviews on at least one vendor website were included. We selected a final total of 11 formulated 25(b) exempt products, which are listed in Table 1.

**Table 1:** List of the tested 25(b) exempt minimum risk repellent products labeled for ticks

Product name	25 (b) Exempt active ingredients included	Manufacturer
Buzz Away Extreme^©^	Castor oil (8%), geranium oil (6%), soybean oil (3%), cedarwood oil (1.5%), citronella oil (1%), peppermint oil (0.5%), lemongrass oil (0.25%)	Quantum® Health
Tickshield	Cedarwood oil (10%), soybean oil (10%)	Cedarcide®
Insect Repellent Cedarwood	Cedarwood oil (5.7%), sodium lauryl sulfate (2.2%), sesame oil (0.1%)	Wondericide®
Insect Repellent Lemongrass	Cedarwood oil (4.2%), sodium lauryl sulfate (2.2%), lemongrass oil (1.5%), sesame oil (0.1%)	Wondericide®
Insect Repellent Peppermint	Cedarwood oil (4.2%), sodium lauryl sulfate (2.2%), peppermint oil (1.5%), sesame oil (0.1%)	Wondericide®
Insect Repellent Rosemary	Cedarwood oil (4.2%), sodium lauryl sulfate (2.2%), rosemary oil (1.5%), sesame oil (0.1%)	Wondericide®
Tickwise Insect Repellent	Cedarwood oil (3.65%), geranium oil (2.64%), citronella oil (1.59%), peppermint oil (0.85%), lemongrass oil (0.3%), rosemary oil (0.3%)	3 Moms Organics LLC
Extra Strength Tick Repellent	Clove oil (2%), geranium oil (2%), peppermint oil (2%), rosemary oil (2%), cedarwood oil (1%), spearmint oil (1%), cinnamon oil (0.5%)	Nantucket Spider®
Natural Tick Repellent	Corn oil (20%), soybean oil (12%), lemongrass oil (1.75%), geraniol (1.75%), citronella oil (0.95%), clove oil (0.5%)	Maggie’s Farm™
All-Natural Tick Repellent	Geraniol (4.75%), sodium lauryl sulfate (1.45%), lemongrass oil (0.2%), peppermint oil (0.05%)	Grandpa Gus’s®
Tick Ban®	Soybean oil (5.2%), castor oil (3%), cedarwood oil (2%), peppermint oil (1.6%), rosemary oil (1.5%), geranium oil (0.6%), lemongrass oil (0.6%), thyme oil (0.5%)	YAYA Organics®

We sought to replicate trials by testing products obtained from different production batches; however, not all repellent products purchased included stock numbers on their bottles; we purchased multiple containers of each product from more than one vendor to increase odds they were derived from multiple production batches. This was done to allow us to determine the consistency of efficacy between different bottles or stocks of the same repellent product. In addition to the 25(b) exempt products, we included positive controls in the form of two EPA-registered DEET-based formulations: OFF!® Deep Woods® (SC Johnson, Racine, WI, USA) and Repel® sportsman formula (Spectrum Brands Inc., Madison, WI, USA). Both were aerosolized spray formulations with a DEET concentration of 25%. Analytical grade ethanol was used as the negative control.

### Repellency bioassay description

We used a finger bioassay, as described previously by Burtis et al. [10], to test all of the formulated products when applied to human skin and to collect negative control data. A single human subject was used to avoid potential variation in repellency among subjects due to differences in the characteristics of their skin. The finger bioassay has been found to yield similar results to the EPA-recommended forearm skin bioassay when using *I. scapularis* nymphs [10], and the finger bioassay is faster and easier to run as it requires movement over a shorter distance by the nymphs and the ticks are more readily observed on all sides of the finger compared to a forearm. Nymphs were accessible to us in large numbers, so it was feasible to use naïve nymphs for each examined timepoint after repellent application.

Prior to treatment, fingers were washed with unscented soap and then washed with ethanol as suggested in EPA guidance [13]. For the finger bioassay, the area between the first and second knuckle of an index finger was treated with 38 µl of the formulation being tested using a micropipette. The formulation was given 30 min to dry or absorb into the skin before repellency testing began. During testing, five nymphs were placed on the nail bed and given 80 s to crawl into or through the treated area on the vertically oriented finger. The same procedure described above was followed for the negative control (ethanol treatment) as the formulated products. Those which entered or crawled through the treated area within this time frame were counted as ‘not repelled.’ Those staying below the first knuckle were considered ‘repelled.’ During the trial for each formulation, both index fingers (left and right) were used, with one stock of each formulation applied to either the left or right finger. Repellency was tested at seven timepoints after the repellent products were applied to the finger (0.5, 1.5, 2.5, 3.5, 4.5, 5.5, and 6.5 h). Six sets of five nymphs were tested at each timepoint for a total of 30 nymphs for each product at each timepoint. All repellency data were corrected using the Abbott correction [14] using the negative control (ethanol treatment) data. Prior to each trial, nymphal behavior was evaluated to ensure they would crawl into or through the treated area within 80 s when no treatment was present. All nymphs which failed to exhibit this behavior were discarded and replaced with more active nymphs.

### Data analyses

Repellency data were analyzed using six logistic regressions for proportions. These were coded as generalized linear mixed models using the glm() command in R with the logit link function. The response was a proportional variable where repellency was coded as the number of ticks repelled over the total number of ticks in a single bioassay (i.e. replicate), which was five cases in all. Our tests were powered to detect a 31% difference at each timepoint at *α* = 0.05 as determined using a two-sided test for proportions. Statistical analyses were conducted using R version 4.2.1 [15].

The first logistic regression for proportions analysis was used to determine whether tick behavior differed significantly between those placed on the left or right finger. The negative control (ethanol treatment) data were used in this analysis, with timepoint (0.5–6.5 h) and finger (left or right) included as predictors. This was necessary as ‘finger’ overlapped with the two different stocks that were used for each formulation, and we needed to determine whether tick behavior differed when placed on one finger versus the other. The second logistic regression for proportions was used to compare the two positive control DEET-based formulations and determine whether their efficacy varied over the 6-h assessment time frame. The third logistic regression for proportions was used to compare repellency among the 11 formulated 25(b) exempt products. Treatment (formulated product), timepoint (0.5–6.5 h) and formulation stock were included as predictors for the second and third logistic regression models. Negative control (ethanol treatment) data were not included in these analyses as these data were used for the Abbott correction applied to the proportion repelled across all 25(b) exempt and DEET-based products that were tested. The Abbott corrected proportion of *I. scapularis* nymphs repelled was used for all analyses, except the first.

The final three logistic regressions for proportions were used to evaluate the repellent effect of the 11 25(b) exempt active ingredients against the positive (DEET) and negative (ethanol) controls at specific timepoints. Repellency dropped significantly after the 1.5 h mark, so the first two timepoints (0.5 and 1.5 h) were compared against the DEET-based products, while the other five timepoints (2.5–6.5 h) were compared against ETOH. Two models were used to compare the repellent effect of the 25(b) exempt products with that of DEET-based products, one at the 0.5 h and another at the 1.5 h timepoint. Both of these analyses only included treatment (formulated product) as predictors. The final logistic regression was used to compare the remaining timepoints (2.5–6.5 h) against ethanol. The model included treatment and timepoint as predictors. A post hoc Tukey test for multiple comparisons was conducted for each of these three analyses so that products could be compared directly against the positive and negative controls.

## Results

**Fig. 1:**
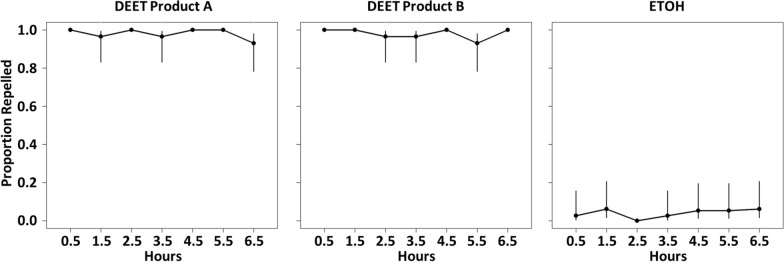
Point estimates and 95% Wilson scores for the proportion of *Ixodes scapularis* nymphs repelled at each timepoint (0.5–6.5 h after application) for the two positive control DEET-based formulated products (**A**, **B**) and the negative control (ETOH). The DEET concentration was 25% for both products

Out of a total of 3194 nymphs used, 254 (8%) were discarded before formulation evaluations began because they failed to crawl into or through the treated area when no treatment was present. Notably, inactive or sluggish nymphs were removed at multiple steps during the rearing process. The proportion reported as discarded above may therefore be lower than expected. In the subsequent negative control (ethanol treatment) trials, 5% of ticks failed to crawl into or through the treated area. There was no significant difference in this behavior in the negative control trials when the left or right finger was used (df = 1,34; deviance residual = 5.53, *P* = 0.80). No reduction in repellency was observed over time from 0.5 to 6.5 h after application, for the two DEET-based products across timepoints (df = 6,77; deviance residual = 6.93, *P* = 0.97). Across the two DEET products, the percent repelled was 100% at 0.5 h, 98% at 1.5 h, 98% at 2.5 h, 97% at 3.5 h, 100% at 4.5 h, 97% at 5.5 h and 97% at 6.5 h. There was no significant difference in the repellent efficacy of the two formulations (df = 1,76; deviance residual = 6.93, *P* = 0.99) or the two stocks used for each formulation (df = 1,75; deviance residual = 6.82, *P* = 0.74). Across all timepoints, both DEET-based formulations repelled 98% of nymphs (Fig. 1).

**Fig. 2:**
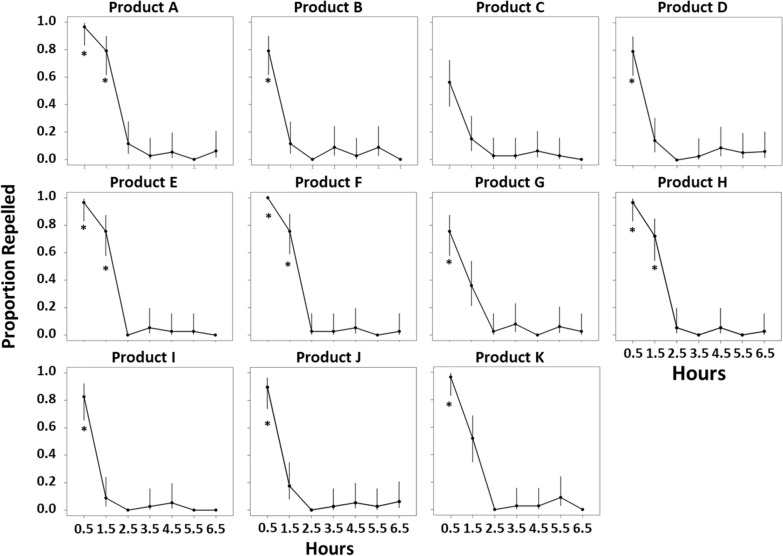
Point estimates and 95% Wilson scores for the proportion of *Ixodes scapularis* nymphs repelled at each timepoint (0.5–6.5 h) after application for the 11 formulated 25(b) exempt repellent products labeled for ticks (**A**–**K**). Timepoints with an asterisk indicate that no significant difference was detected between the effectiveness of that product and the two DEET-based products (25% concentration) tested at that time point according to the post hoc Tukey tests. The active ingredients with the highest concentrations in each product were: Product **A** (cedarwood oil: 4.2%), Product **B** (cedarwood oil: 4.2%), Product **C** (cedarwood oil: 4.2%), Product **D** (cedarwood oil: 5.7%), Product **E** (geraniol: 4.75%), Product **F** (soybean oil: 5.2%), Product **G** (castor oil: 8%), Product **H** (corn oil: 20%), Product **I** (cedarwood oil: 3.65%), Product **J** (cedarwood oil: 10%), Product **K** (clove oil: 2%)

Across the 11 25(b) exempt products and all timepoints, there were significant differences between the formulations (df = 10,445; deviance residual = 91.2; *P* = 0.029). The overall repellent efficacy dropped significantly over time after application for all formulations (df = 6, 455; deviance residual = 111.2; *P* < 0.001). Across all the 11 25(b) exempt products tested, repellency was 86% at 0.5 h, fell to 40% at 1.5 h and fell again to 2% at 2.5 h (Fig. 2). The overall percent nymphs repelled across all 11 25(b) exempt products for the remaining timepoints (3.5, 4.5, 5.5, and 6.5 h) was 4% (Fig. 2). Repellent efficacy did not differ significantly between the two stocks tested for any formulation (df = 1,444; deviance residual = 90.7; *P* = 0.505).

Comparing the 11 25(b) exempt products against the two DEET-based products at the 0.5 h timepoint, only one (Product C) showed significantly lower repellency than the DEET-based products. At the 1.5 h mark, seven of the 25(b) exempt products (B, C, D, G, I, J, and K) repelled significantly fewer ticks than the DEET-based products, whereas the other four products (A, E, F, and H) performed similarly to the DEET-based products (Fig. 2). At timepoints ≥ 2.5 h, the repellency did not differ significantly from the negative control (ethanol) treatment for any of the 11 25(b) exempt products.

## Discussion

None of the 11 formulated repellent products that utilized 25(b) exempt active ingredients showed significantly higher repellency for *I. scapularis* nymphs than the negative control ethanol treatment past the 1.5 h timepoint in our evaluation. By contrast, both DEET-based products remained effective in repelling ticks (98% repelled) for the entire 6.5-h period. While we observed qualitatively lower repellency compared with DEET, ten of the 25(b) exempt products showed statistically similar repellency compared with the DEET-based products at 0.5 h post-application; only four did not differ significantly from the two DEET-based products at 1.5 h (Fig. 2). Notably, our statistical tests were powered to detect a roughly 30% difference in repellency at each timepoint. A lack of statistical power to detect a difference does not necessarily equate to equivalent efficacy. Only six of the 25(b) exempt products showed ≥ 90% repellency for *I. scapularis* nymphs at the 0.5 h mark, and none hit that threshold at the 1.5 h timepoint. Further testing of the most efficacious products may help provide some guidance for re-application regimes for these products to allow for similar protection to that of DEET-based products.

The user guidance on the labels of the 25(b) exempt products varied, with some providing specific application instructions, while others did not. Of the 11 products tested, six specifically mentioned that the product should be re-applied every 1 or 2 h. Two made specific claims about the product being effective for ≥ 6 h, while three provided no specific timeframe for the duration of repellent effects. The repellent effect of those two products claiming to be effective for ≥ 6 h was not found to last longer than the other products tested. It is also notable that three of the 25(b) exempt products did not list lot numbers on the bottle and seven failed to list expiration dates. The inconsistency across labels made it difficult to determine how these products should be used, and the lack of expiration dates or lot numbers on many made it difficult to evaluate how long the product was on the shelf prior to our testing.

Overall, our results align closely with those of previous studies evaluating the repellent activity of unformulated 25(b) exempt active ingredients against *I. scapularis* nymphs and adults in human skin bioassays. These previous studies found variation in the duration of efficacy between different active ingredients, but none of them were highly effective for > 2 h [9, 10]. Notably, results were similar between those two studies, with limited repellency past 1 h for peppermint oil and low overall repellency for rosemary oil, despite the use of different concentrations (10% versus 20%) and carriers (lotion versus ethanol). Many of the commercial formulations we tested used relatively low concentrations of the 25(b) exempt active ingredients. Only two used concentrations ≥ 10% of any individual active ingredient (Table 1). Despite this, we observed similar results to previous repellency tests that used unformulated 25(b) exempt active ingredients, with repellency decreasing after the first hour of application. Additionally, there was no indication that the more effective products used higher overall concentrations of active ingredients, specific individual 25(b) exempt active ingredients or specific combinations of 25(b) exempt active ingredients. More study is needed, but it is possible that the repellent activity of these active ingredients is not strongly linked with concentration above a certain threshold.

Despite evidence that the repellency of unformulated 25(b) exempt active ingredients, as well as formulated products, drops sharply after 2 h when applied to human skin, previous studies indicated that one formulated 25(b) exempt product remained effective as a repellent against *I. scapularis* for longer than we observed when it was applied to textiles [16, 17]. Two studies evaluated a formulated product called EcoSMART Organic Insect Repellent (1% geranol, 0.5% cinnamon oil, 0.5% lemongrass oil, and 0.5% rosemary oil). Both studies found the product was effective (> 90% repellency) for ≥ 48 h after application [16, 17]. However, the methodology of these studies differed from our evaluation in several ways: repellents were applied to textiles (drag cloth and overalls) rather than human skin, the ticks made initial contact with a repellent-treated surface rather than being introduced onto a nontreated surface and then approaching a repellent surface, and repellency was defined by ticks dislodging from a repellent-treated surface rather than avoiding to move onto a repellent-treated surface. It is possible that the methodology used in these studies could detect a weaker repellent effect than our human skin bioassay. It is also possible that the ticks are more easily repelled from treated textiles compared to treated skin, as the skin itself provides a powerful stimulus for the ticks to continue moving toward a bite site despite the presence of a repellent. None of the 25(b) exempt products we examined provided specific guidance on their label for how often reapplication is needed depending upon where the repellent is applied (skin versus clothing).

Two recent surveys of the public indicated that many respondents are willing to use both ‘natural’ and ‘synthetic’ repellent products [18, 19], but neither survey contained specific language regarding EPA-registered versus 25(b) exempt products. There is an apparent market for minimum risk 25(b) repellent products containing only compounds considered by the EPA to be safe for use on human skin; however, the drivers behind their selection or preference over EPA-registered products that might be considered ‘natural’ (e.g. oil of lemon eucalyptus) are unknown. The 25(b) exempt repellent products tested in this study were not as effective for as long as those which were DEET-based, but some did show at least 70% repellency for short time periods (≤ 1.5 h). Given that there is a market for 25(b) exempt repellent products, further research and evaluation of potential stabilizers and synergists for these products may help improve their ability to provide longer lasting protection.

## Conclusions

Neither﻿ the claims on the labels nor specific active ingredients and their concentrations appeared to predict the duration of efficacy we observed for the 25(b) exempt products. These products are not registered with the EPA, so the methods used to determine the application guidelines on their labels are unclear. Consumers should be aware that both the level of efficacy and the duration of repellency may differ among unregulated 25(b) exempt repellent products labeled for use against ticks. We encourage more research on these products and the 25(b) exempt active ingredients they contain to help determine and improve their efficacy as repellents under different conditions.

## Data Availability

The data supporting the findings of the study must be available within the article and/or its supplementary materials, or deposited in a publicly available database.
